# Combined effect of obesity and uric acid on nonalcoholic fatty liver disease and hypertriglyceridemia

**DOI:** 10.1097/MD.0000000000006381

**Published:** 2017-03-24

**Authors:** Shujun Zhang, Tingting Du, Mengni Li, Huiming Lu, Xuan Lin, Xuefeng Yu

**Affiliations:** a Division of Endocrinology, Department of Internal Medicine, Tongji Hospital, Tongji Medical College, Huazhong University of Science and Technology; b Department of Health Examination; c Department of Endocrinology, Wuhan Iron and Steel Company (WISCO) General Hospital, Wuhan, Hubei province, China.

**Keywords:** insulin resistance, interaction, metabolic syndrome

## Abstract

Hyperuricemia is associated with metabolic syndrome (MetS), but the association is often confounded by the shared background of obesity. We sought to explore the modifying effects of obesity on the association between uric acid (UA), MetS components, and nonalcoholic fatty liver disease (NAFLD).

We conducted a cross-sectional study in a Chinese population of 10,069 participants aged ≥20 years. Multiplicative interaction between obesity (BMI ≥25 kg/m^2^) and elevated UA was assessed using an interaction term in a logistic regression analysis. The presence of additive interaction was assessed based on the relative excess risk due to the interaction (RERI) and the attributable proportion due to the interaction (AP).

There was no evidence of a multiplicative interaction between obesity and elevated UA on MetS components and NAFLD. However, there was a strong additive interaction between obesity and elevated UA with regard to NAFLD (RERI of 6.47 [95% CI 3.42–9.53] for men and 5.87 [1.55–10.19] for women) and hypertriglyceridemia (RERI of 1.38 [0.57–2.20] for men and 1.38 [0.08–2.67] for women). In addition, 42% and 36% of the increased odds of NAFLD for men and women, respectively, can be explained by an interaction between obesity and elevated UA (AP of 0.42 [95% CI (0.30–0.54)] for men and 0.36 [0.17–0.55] for women). Similarly, the interaction accounted for 27% and 26% of the increased risk of hypertriglyceridemia for men and women (AP of 0.27 [0.14–0.41] for men and 0.26 [0.06–0.47] for women).

In this population, obesity and elevated UA synergistically interacted to increase the risk of NAFLD and hypertriglyceridemia.

## 1 Introduction

Metabolic syndrome (MetS) is a cluster of interrelated cardiometabolic risk factors^[1]^ that are associated with an increased risk of type 2 diabetes and cardiovascular diseases. The prevalence of MetS dramatically increased worldwide, ranging from 10% to 84% depending on the age, gender, and ethnicity/race of the population.^[2]^ The studies performed in China indicate it is experiencing an epidemic of MetS, with a high prevalence of 33.9% among general Chinese population.^[3]^ The pathogenesis of MetS remains unclear, although the possible involvement of insulin resistance has been implicated as a linking factor. Determination of the risk factors of MetS is essential for identification and intervention of MetS.

Serum levels of uric acid (UA), the end-product of purine metabolism, are maintained by a balance between the production and excretion of UA.^[4]^ Increasing evidence indicates that elevated UA levels, even within the normal range, are related to MetS.^[5]^ Cardiovascular disease is a major comorbidity of hyperuricemia^[6]^; however, whether elevated UA levels are independently associated with cardiovascular disease risk is still controversial. It is likely that the relative importance of UA, per se, as a cardiovascular disease risk factor may involve an interaction with the other metabolic disorders.^[7]^

Obesity is a global public health problem and is associated with MetS and hyperuricemia.^[8]^ With the rapid socio-economic growth, the prevalence of obesity has increased rapidly among Chinese adults during the past decades, from 2.9% and 5.0% to 11.4% and 10.1% among men and women, respectively.^[9]^ A shared background of obesity usually confounds the relationship observed between UA and MetS. Several studies have investigated the role of obesity in the association of UA and MetS, but the results were inconsistent.^[10–12]^ The possible interaction between obesity and UA with regard to certain components of MetS has not been well studied. In addition, nonalcoholic fatty liver disease (NAFLD), which is characterized by excessive fat accumulation in the liver without excessive alcohol consumption, has been regarded as the hepatic manifestation of MetS.^[13]^ NAFLD has frequently been associated with many metabolic abnormalities, such as insulin resistance, obesity, type 2 diabetes mellitus, and hyperlipidemia, which are the main features of MetS.^[14]^ It has been reported that approximately 90% of the NAFLD patients present more than 1 component of MetS, and about 33% of the patients meet the criteria of MetS.^[15]^ Recent studies have demonstrated a close relationship between serum UA and NAFLD.^[16–18]^ However, few studies have explored whether obesity, as a major risk factor for NAFLD, can modify the association between UA and NAFLD.

In the present study, we sought to explore the modifying effect of obesity on the association between elevated UA, MetS components, and NAFLD in a Chinese population.

## 2 Methods

### 2.1 Subjects and study design

The participants in the present study were from the Wuhan Iron and Steel Company (WISCO), consisting of Chinese employee aged ≥20 years. The data derived from a health examination of all of the employees and retirees at the WISCO General Hospital in 2009.^[19–21]^ Questionnaires were used to collect data regarding demographic characteristics, including age, sex, medical history, and drinking status. We excluded individuals who were taking medicines for diabetes, hypertension, dyslipidemia, or hyperuricemia; who had missing information on their age, sex, body mass index (BMI), fasting plasma glucose (FPG), blood pressure (BP), triglycerides (TG), high density lipoprotein-cholesterol (HDL-C), UA or liver ultrasonography data; and who had chronic kidney disease (defined as an estimated glomerular filtration rate <60 mL/min per 1.73 m^2^). Finally, 10,069 participants, 6378 males and 3691 females, were included in the present study. The fact that male participants accounted for 63.3% of the entire cohort was consistent with the sex percentage at WISCO. Our study was approved by the Institutional Review Board of the WISCO General Hospital, and the informed consent requirement was exempted because of our retrospective estimation of a de-identified database.

### 2.2 Anthropometric and biochemical measurements

Physical examination was performed and anthropometric parameters were obtained including weight, height, and BP. Weight was measured with the participants wearing light clothing and height was measured without shoes. BMI was calculated as weight (in kilograms)/height square (in meters). BP was measured twice every 5 minutes on the right arm after 5 minutes of rest with the participants seated. The mean of the 2 measures was taken for data analysis.

Blood samples were collected from the antecubital vein of all participants after fasting at least 10 hours overnight and biochemically analyzed for FPG, UA, creatinine, alanine aminotransferase, TG, and HDL-C levels. All the measurements were determined using an auto-analyzer (Hitachi 7600, Ltd, Tokyo, Japan). The triglycerides and glucose index (TyG) were calculated using the published formula:^[22]^ Ln [TG (mg/dL) × FPG (mg/dL)/2].

### 2.3 Definitions

Given that the UA levels differed substantially by gender, sex-specific quartiles of UA levels were established (for men, first quartile: ≤4.8 mg/dL, second quartile: 4.9–5.5 mg/dL, third quartile: 5.6–6.3 mg/dL, fourth quartile: ≥6.4 mg/dL; for women, corresponding cut-points: ≤3.5 mg/dL, 3.6–4.0 mg/dL, 4.1–4.8 mg/dL, ≥4.9 mg/dL). We defined an elevated UA level as the highest UA quartile (≥6.4 mg/dL in men and ≥4.9 mg/dL in women), and a normal UA level as the lower 3 quartiles.

According to the World Health Organization (WHO) expert consulate for Asians,^[23]^ obese status is defined as a BMI ≥25 kg/m^2^ and nonobese status is a BMI <25 kg/m^2^.

Components of MetS are defined based on the following definitions of the Adult Treatment Panel-III (ATP III):^[24]^ (1) abdominal obesity: waist circumference ≥90/80 cm for men/women); (2) elevated BP: systolic/diastolic BP ≥130/85 mm Hg; (3) elevated FPG: FPG ≥5.6 mmol/L (100 mg/dL); (4) elevated TG: TG ≥1.7 mmol/L (150 mg/dL); and (5) low HDL-C: HDL-C ≤1.0/1.3 mmol/L (40/50 mg/dL) for men/women. Of these components, we assessed the associations between elevated BP, elevated FPG, elevated TG, and low HDL-C with UA levels in obese and nonobese subjects. As waist measurement was not available for the study population, a BMI of ≥25 kg/m^2^ for all patients was substituted as an index of obesity which was taken as a component of MetS. Previous studies have confirmed the validation of this definition.^[25]^

As NAFLD was regarded as a hepatic manifestation of metabolic syndrome, we also evaluated the relationship between NAFLD with UA levels in obese and nonobese subjects.

According to the guideline of the Asia-Pacific Working Party,^[26]^ NAFLD is diagnosed based on the presence of fatty liver, as assessed using ultrasonography, ruling out excessive alcohol intake (>140 g/wk for men, >70 g/wk for women), hepatic virus infection, or the use of steatogenic or hepatotoxicity medications.

### 2.4 Statistical analysis

All statistical analyses were conducted using SPSS (version 20.0, Chicago, IL). Men and women were both stratified into 4 mutually exclusive groups based on BMI and UA levels, namely, nonobese with normal UA or elevated UA level and obese with normal UA or elevated UA level. The basic characteristics of the participants were presented as a median and interquartile range for continuous variables and as a percentage for categorical variables. Differences between any 2 groups were assessed using the Mann–Whitney *U* test or chi-square test. The age-adjusted association between UA and MetS components was determined using logistic regression analysis. The multiplicative interaction between BMI and UA was assessed using a cross-product interaction term included in the logistic regression model.

The additive interaction may better reflect a biological interaction.^[27]^ Therefore, we used a method proposed by Rothman^[28]^ to test for additive interaction between BMI and UA levels. To quantify the amount of additive interaction, we calculated 2 measures using the approach of Andersson et al,^[29]^ namely, the relative excess risk due to the interaction (RERI) and the attributable proportion due to the interaction (AP). An RERI and AP of zero indicate the absence of an additive interaction.

A 2-tailed *P*-value <0.05 was considered to be significant.

## 3 Results

### 3.1 Characteristics of the study subjects

The average age and BMI of this study were 49.5 ± 14.7 years and 23.6 ± 3.1 kg/m^2^, respectively. Clinical characteristics of the population stratified by BMI and UA levels are presented in Table 1. For men, individuals with elevated levels of UA were more likely to have an adverse metabolic risk profile, including higher BMI, systolic/diastolic BP, TG, TG/HDL-C, TyG, and ALT, and lower HDL-C (all *P* < 0.01), compared with those with normal UA level for both the nonobese and obese groups. The difference in level of FPG between the elevated and normal UA group was statistically significant for nonobese men, but not for obese men. For women, subjects with high levels of UA were older than those with normal UA, and had significantly higher BMI, systolic BP, FPG, TG, TG/HDL-C, TyG, and ALT, and lower HDL-C (all *P* < 0.02), regardless of their obesity status.

**Table 1:**
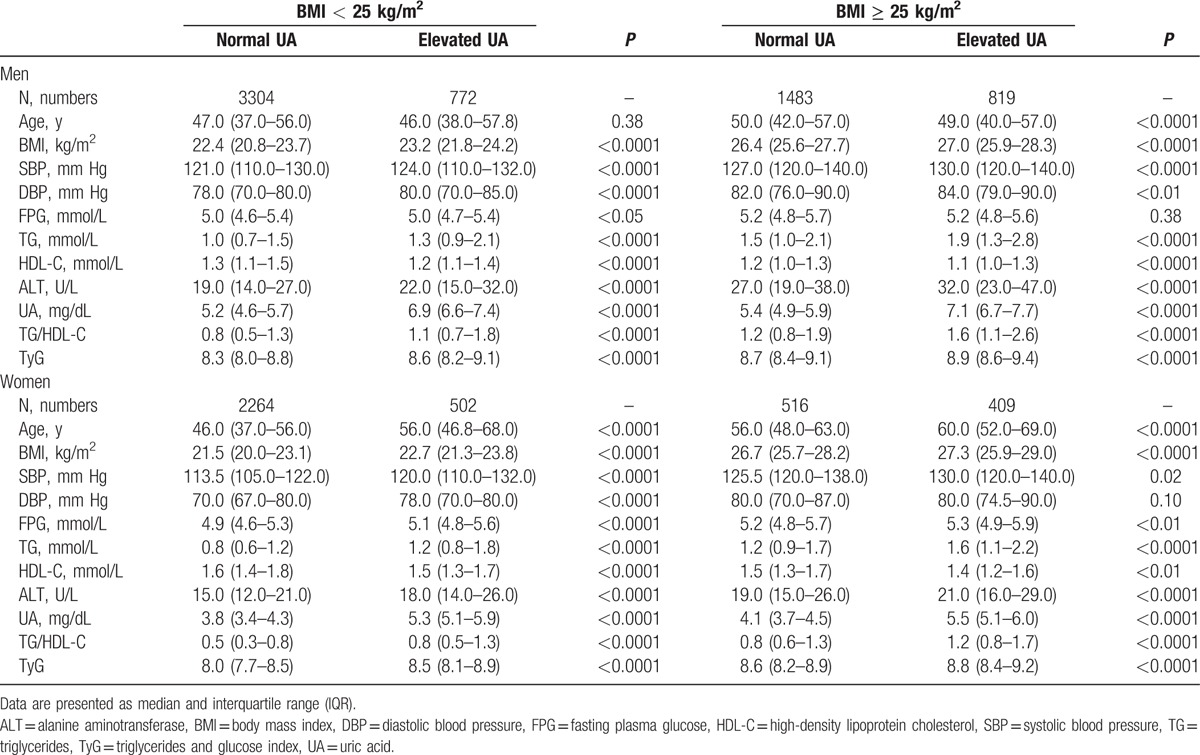
Clinical characteristics of the study population.

The TyG index was significantly elevated in MetS and NAFLD participants for men (MetS 9.4 ± 0.6, non-MetS 8.4 ± 0.6, *P* < 0.0001; NAFLD 8.9 ± 0.7, non-NAFLD 8.3 ± 0.6, *P* < 0.0001) and women (MetS 9.2 ± 0.6, non-MetS 8.2 ± 0.6, *P* < 0.0001; NAFLD 8.8 ± 0.6, non-NAFLD 8.1 ± 0.6, *P* < 0.0001), as compared with their counterparts. For men, the OR and 95% CI of TyG for MetS and NAFLD was 12.5 (10.8–14.6) and 5.3 (4.8–5.9), respectively. For women, the corresponding figures were 16.2 (12.6–20.7) and 6.5 (5.6–7.5), respectively.

### 3.2 Associations between UA and metabolic disorders according to BMI

The prevalence of MetS components in UA groups according to sex and BMI is shown in Table 2. For men, regardless of obesity status, the prevalence of elevated BP, elevated TG, low HDL-C, and NAFLD were significantly higher in the elevated UA group than in the normal UA group (all *P* < 0.01), but not an elevated FPG. For women, the prevalence of all evaluated components of MetS and NAFLD were higher among those with an elevated UA level compared to those with a normal UA level in both BMI groups, albeit the prevalence of a low HDL-C was marginally different between individuals with an elevated or normal UA level in the obese group (*P* = 0.07).

**Table 2:**
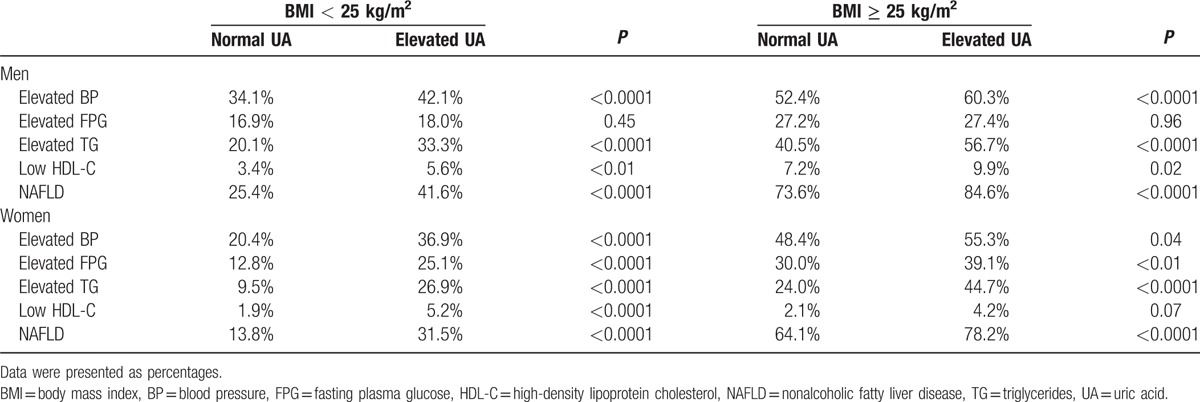
Prevalence of metabolic syndrome components and nonalcoholic fatty liver disease in groups stratified by the body mass index and uric acid levels.

The age-adjusted ORs for MetS components and NAFLD are shown in Table 3. The *P*-value of the interaction term was used to evaluate the multiplicative interaction. Compared with nonobese individuals with a normal UA level, obese subjects with an elevated UA level had a significantly increased risk of MetS components and NAFLD in both men and women. In particular, ORs for NAFLD (15.49 [95% CI 12.57–19.09] for men and 16.25 [12.39–21.31] for women) and hypertriglyceridemia (5.05 [4.27–5.96] for men and 5.25 [4.07–6.77] for women) were significantly greater than other MetS components in obese subjects with elevated UA levels. However, there was no evidence of a multiplicative interaction between obesity and UA for increasing the risk of these assessed metabolic disorders.

**Table 3:**
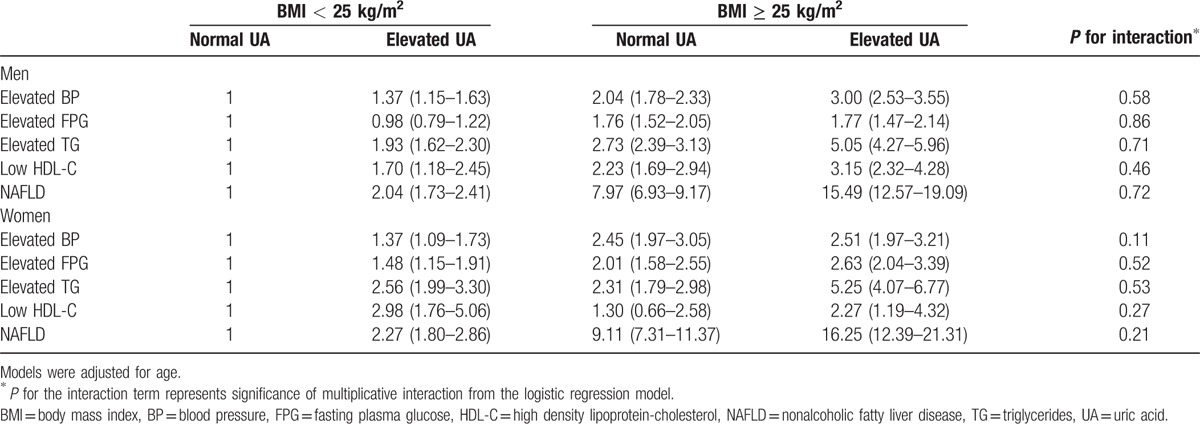
ORs for the association between serum levels of uric acid and metabolic syndrome components or nonalcoholic fatty liver disease stratified by the body mass index.

### 3.3 Additive interaction between obesity and UA in association with MetS components and NAFLD

**Figure 1:**
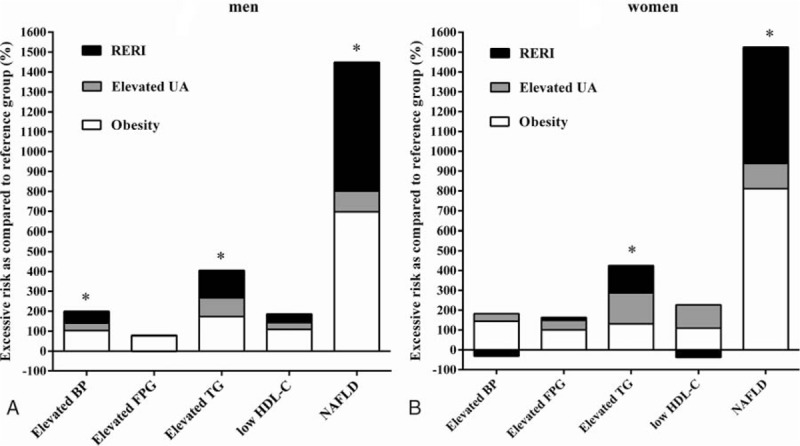
Additive interaction between obesity and elevated UA levels for MetS components and NAFLD in men (A) and women (B). ∗ indicates *P*-value <0.05. BP = blood pressure, FPG = fasting plasma glucose, HDL-C = high-density lipoprotein cholesterol, MetS = metabolic syndrome, NAFLD = nonalcoholic fatty liver disease, RERI = the relative excess risk due to the interaction, TG = triglycerides, UA = uric acid.

An additive interaction better reflects a biological interaction.^[27]^ Therefore, we further conducted an additive interaction analysis to detect whether the combined influence of obesity and UA on the risk of MetS components exceeded the sum of their individual impact. The results are presented in Table 4 and Fig. 1. There was a strong additive interaction between obesity and elevated UA levels on the risk of NAFLD (RERI of 6.47 [95% CI 3.42–9.53] for men and 5.87 [1.55–10.19] for women). In other words, the concurrence of obesity and elevated UA levels conferred a 6.47- and 5.87-fold relative excess risk for men and women, respectively, beyond the sum of the individual risks for obesity and elevated UA levels. Similarly, the RERI for hypertriglyceridemia was statistically significant in men (RERI of 1.38 [95% CI 0.57–2.20]) and women (RERI 1.38 [0.08–2.67]). A relatively weak additive interaction was detected for the risk of an elevated BP in men (RERI 0.59 [0.04–1.14]). There was no evidence of an additive interaction between obesity and elevated UA for the risk of elevated FPG and low HDL-C, and elevated BP in women (RERI ≈ 0 and AP ≈ 0).

**Table 4:**
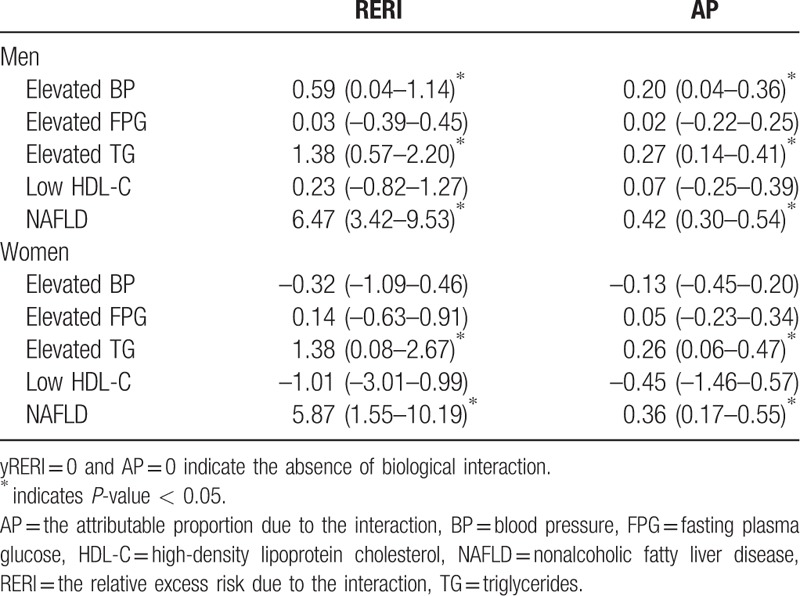
Assessment for the biological interaction between obesity and elevated levels of uric acid.

## 4 Discussion

In the present study, we found evidence that being obese modified the association of UA with NAFLD and hypertriglyceridemia. The magnitude of the effect of modification was large; obese men and women with elevated UA had 15.49 and 16.25 times the risk of having NAFLD, and 5.05 and 5.25 times the risk for hypertriglyceridemia, which were much higher than the increased risks among obese individuals with normal UA levels or nonobese persons with elevated UA levels. These findings suggest a synergistic effect of obesity and an elevated UA level for increasing the risk of NAFLD and hypertriglyceridemia. No statistically significant interaction was observed with regard to other MetS components.

Obesity is a common condition related to hyperuricemia and MetS components. However, the modifying effect of obesity on the association between UA with MetS and NAFLD has not been well studied. Several lines of evidence suggest that obesity could modify the relationship between UA and MetS, but there is still a plenty of controversy. A large-sample study of European participants suggested that elevated BMI is an essential confounding factor to the observational relationship analyses of UA and related conditions.^[30]^ A recent study from Norway conducted by Norvik et al^[10]^ also demonstrated that BMI could modify the association of UA and some MetS components. They observed that elevated UA predicted the onset of elevated BP and elevated FPG in subjects with a BMI >25 kg/m^2^, but not in those with a BMI < 25 kg/m^2^, but this was not the case for other MetS components. In contrast, another study in a general population from Korea^[11]^ reported that UA was associated with an increased risk of MetS in a subgroup with a BMI <25 kg/m^2^, but not in a subgroup with a BMI >25 kg/m^2^. This discrepancy may be due to differences in study design and the study population. In addition, a study of a prospective cohort of American Indians with a high prevalence of obesity suggests that UA levels do not predict MetS when taking fat-free mass into account, indicating that the body composition that is altered in the context of obesity has an important role in the relationship between UA and MetS.^[12]^ This finding contradicts the evidence from a study conducted in Korea^[11]^ that showed that the relationship between UA and MetS remained significant after adjusting for multiple confounders, including body composition. The difference in the obesity status of the 2 cohorts is likely to be a major factor responsible for the discordant results. Taken together, these findings suggest that obesity may be an essential confounder in the relation between UA and metabolic disorders, although some may disagree.

Our findings show that obesity and elevated UA levels have a pronounced synergistic effect on the development of NAFLD and hypertriglyceridemia. NAFLD is widely regarded as a liver manifestation of MetS and is also closely related to central obesity. Hypertriglyceridemia is a major metabolic abnormality associated with the presence of NAFLD.^[21]^ Furthermore, we observed in this study that the TyG index, including lipid parameter and proposed as a surrogate marker for insulin resistance,^[22,31]^ was increased in both MetS and NAFLD patients and was associated with a higher risk for the diseases, which indicating the close relationship between lipid metabolism, insulin resistance, and NAFLD. In accordance, the association between TyG and fatty liver has also been demonstrated in previous studies.^[32,33]^ The combined effect of obesity and elevated UA levels on the risk of NAFLD and hypertriglyceridemia may share a common pathogenesis. Obesity could result in elevated UA levels through a reduction in urinary urate excretion and overproduction of UA. Notably, visceral adiposity is more tightly linked to overproduction of UA than subcutaneous fat.^[34]^ In turn, UA becomes a strong pro-oxidant in the presence of obesity.^[35]^ Hyperuricemia induces alternations in oxidative homeostasis in adipocytes, including a decrease in nitric oxide bioavailability and an increase in lipid oxidation, which may play an important role in the subsequent development of hepatic steatosis and hypertriglyceridemia.^[36]^ In addition, recent studies indicate that fructose is associated with hepatic steatosis.^[37]^ In addition to directly generating triglycerides, fructose also stimulates triglycerides synthesis via increasing UA production.^[16]^ Hence, fructose may be a crucial link between obesity, elevated UA, NAFLD, and hypertriglyceridemia.

The additive interaction between obesity and UA on hypertriglyceridemia provides a possible explanation for the finding that high levels of UA have a stronger association with hypertriglyceridemia than other MetS components. Previous studies ^[10,38–40]^ have shown that elevated UA levels are associated with MetS components, especially hypertriglyceridemia, elevated BP, and low HDL-C; however, the relationship varied based on the study. This variation, to some extent, was due to the differences in the selected population and specific demographics of the participants. Nonetheless, the relationship between UA and hypertriglyceridemia was strong and stable. The mechanism that links UA to TG has not been elucidated. One experimental study demonstrated that lowering serum UA correlated directly with a reduction of TG levels and hypertriglyceridemia was completely blocked by the decrease of UA levels with allopurinol.^[41]^ Although the role of UA in TG metabolism remains unknown, UA might be implicated in either the reduction of clearance or the overproduction of TGs. Our data provide another possible explanation. Obesity, as a common context of hyperuricemia and MetS and a major risk factor for the development of abnormal TG, may interact with UA and confer an additional risk of hypertriglyceridemia. The biological mechanism underlying this problem needs to be further investigated.

Several important limitations of the present study should be noted. First, due to a cross-sectional design, we could not explore the causality of the associations. Second, we identified NAFLD using only ultrasonography, which is a reasonably accurate method for diagnosing NAFLD in patients with only modest amounts of liver fat (>30% liver fat infiltration). Third, the participants in the present study were from a selected population (industrial employees and retired workers) with a preponderance of men; therefore, we should be cautious in extrapolating the findings to the general Chinese population or to other ethnicities. Nonetheless, the large cohort of the present investigation ensures sufficient power in determining the significance of the interaction between UA and BMI. Fourth, this study did not evaluate the dietary profile of the studied population due to the lack of diet information, which might have influenced UA concentrations. However, high UA levels are mainly due to abnormal metabolism which is revealed that high UA levels are associated with various metabolic risk factors, such as insulin resistance, obesity, type 2 diabetes, hypertension, and dyslipidemia, rather than dietary profile in adults.^[42]^

In a conclusion, obesity and elevated UA levels have a pronounced synergistic effect on the development of NAFLD and hypertriglyceridemia. The clinical significance of our finding is substantial because a large proportion of cases of NAFLD (42% for men and 36% for women) and hypertriglyceridemia (27% for men and 26% for women) can be explained based on an interaction between obesity and elevated levels of UA. This evidence indicates that the burden of NAFLD and hypertriglyceridemia in obese patients may be markedly reduced after improving serum UA levels.

## Acknowledgments

We thank all the participants for their contribution and participation.

## References

[1] Alberti KG Eckel RH Grundy SM Harmonizing the metabolic syndrome: a joint interim statement of the International Diabetes Federation Task Force on Epidemiology and Prevention; National Heart, Lung, and Blood Institute; American Heart Association; World Heart Federation; International Atherosclerosis Society; and International Association for the Study of Obesity. Circulation 2009;120:1640–5. 19805654 10.1161/CIRCULATIONAHA.109.192644

[2] Kaur J A comprehensive review on metabolic syndrome. Cardiol Res Pract 2014;2014:943162. 24711954 10.1155/2014/943162 PMC3966331

[3] Lu J Wang L Li M Metabolic syndrome among adults in China—The 2010 China Noncommunicable Disease Surveillance. J Clin Endocrinol Metab 2017;102:507–15. 10.1210/jc.2016-2477 27898293

[4] Mandal AK Mount DB The molecular physiology of uric acid homeostasis. Annu Rev Physiol 2015;77:323–45. 25422986 10.1146/annurev-physiol-021113-170343

[5] Yuan H Yu C Li X Serum uric acid levels and risk of metabolic syndrome: a dose-response meta-analysis of prospective studies. J Clin Endocrinol Metab 2015;100:4198–207. 26308292 10.1210/jc.2015-2527

[6] Zhu Y Pandya BJ Choi HK Comorbidities of gout and hyperuricemia in the US general population: NHANES. Am J Med 2012;125:679–87. e671. 22626509 10.1016/j.amjmed.2011.09.033

[7] Culleton BF Larson MG Kannel WB Serum uric acid and risk for cardiovascular disease and death: the Framingham Heart Study. Ann Intern Med 1999;131:7–13. 10391820 10.7326/0003-4819-131-1-199907060-00003

[8] Kelly T Yang W Chen CS Global burden of obesity in 2005 and projections to 2030. Int J Obes 2008;32:1431–7. 10.1038/ijo.2008.102 18607383

[9] Xi B Liang Y He T Secular trends in the prevalence of general and abdominal obesity among Chinese adults 1993–2009. Obes Rev 2012;13:287–96. 22034908 10.1111/j.1467-789X.2011.00944.x PMC3276709

[10] Norvik JV Storhaug HM Ytrehus K Overweight modifies the longitudinal association between uric acid and some components of the metabolic syndrome: the Tromso Study. BMC Cardiovasc Disord 2016;16:85. 27165776 10.1186/s12872-016-0265-8 PMC4862215

[11] Yu TY Jee JH Bae JC Serum uric acid: a strong and independent predictor of metabolic syndrome after adjusting for body composition. Metabolism 2016;65:432–40. 26975535 10.1016/j.metabol.2015.11.003

[12] Ferrara LA Wang H Umans JG Serum uric acid does not predict incident metabolic syndrome in a population with high prevalence of obesity. Nutr Metab Cardiovasc Dis 2014;24:1360–4. 25063537 10.1016/j.numecd.2014.06.002 PMC4250289

[13] Marchesini G Brizi M Bianchi G Nonalcoholic fatty liver disease: a feature of the metabolic syndrome. Diabetes 2001;50:1844–50. 11473047 10.2337/diabetes.50.8.1844

[14] Abenavoli L Milic N Di Renzo L Metabolic aspects of adult patients with nonalcoholic fatty liver disease. World J Gastroenterol 2016;22:7006–16. 27610012 10.3748/wjg.v22.i31.7006 PMC4988304

[15] Bugianesi E Moscatiello S Ciaravella MF Insulin resistance in nonalcoholic fatty liver disease. Curr Pharm Des 2010;16:1941–51. 20370677 10.2174/138161210791208875

[16] Lanaspa MA Sanchez-Lozada LG Choi YJ Uric acid induces hepatic steatosis by generation of mitochondrial oxidative stress: potential role in fructose-dependent and -independent fatty liver. J Biol Chem 2012;287:40732–44. 23035112 10.1074/jbc.M112.399899 PMC3504786

[17] Li Y Xu C Yu C Association of serum uric acid level with non-alcoholic fatty liver disease: a cross-sectional study. J Hepatol 2009;50:1029–34. 19299029 10.1016/j.jhep.2008.11.021

[18] Wu SJ Zhu GQ Ye BZ Association between sex-specific serum uric acid and non-alcoholic fatty liver disease in Chinese adults: a large population-based study. Medicine (Baltimore) 2015;94:e802. 25929934 10.1097/MD.0000000000000802 PMC4603030

[19] Du T Yu X Yuan G Combined influence of nonalcoholic fatty liver and body size phenotypes on diabetes risk. Cardiovasc Diabetol 2015;14:144. 26511621 10.1186/s12933-015-0306-0 PMC4625438

[20] Du T Sun X Lu H Associations of serum uric acid levels with cardiovascular health factors: differences by sex, age and body mass index in Chinese participants. Eur J Int Med 2014;25:388–93. 10.1016/j.ejim.2014.03.004 24702838

[21] Du T Sun X Yuan G Lipid phenotypes in patients with nonalcoholic fatty liver disease. Metabolism 2016;65:1391–8. 27506745 10.1016/j.metabol.2016.06.006

[22] Guerrero-Romero F Simental-Mendia LE Gonzalez-Ortiz M The product of triglycerides and glucose, a simple measure of insulin sensitivity. Comparison with the euglycemic-hyperinsulinemic clamp. J Clin Endocrinol Metab 2010;95:3347–51. 20484475 10.1210/jc.2010-0288

[23] Consultation WHOE. Appropriate body-mass index for Asian populations and its implications for policy and intervention strategies. Lancet 2004;363:157–63. 14726171 10.1016/S0140-6736(03)15268-3

[24] Expert Panel on Detection, Evaluation, and Treatment of High Blood Cholesterol in Adults. Executive summary of the third report of the National Cholesterol Education Program (NCEP) expert panel on detection, evaluation, and treatment of high blood cholesterol in adults (adult treatment panel III). JAMA 2001;285:2486–97. 11368702 10.1001/jama.285.19.2486

[25] Fukuda Y Hashimoto Y Hamaguchi M Triglycerides to high-density lipoprotein cholesterol ratio is an independent predictor of incident fatty liver; a population-based cohort study. Liver Int 2016;36:713–20. 26444696 10.1111/liv.12977

[26] Farrell GC Chitturi S Lau GK Asia-Pacific Working Party on NAFLD. Guidelines for the assessment and management of non-alcoholic fatty liver disease in the Asia-Pacific region: executive summary. J Gastroenterol Hepatol 2007;22:775–7. 17565629 10.1111/j.1440-1746.2007.05002.x

[27] Rothman KJ Greenland S Lash TL Modern Epidemiology. 3rd edn Philadelphia, PA: Lippincott, Williams & Wilkins; 2008.

[28] Oxford University Press, Rothman KJ Epidemiology: An Introduction. Vol 56. 2002 959.

[29] Andersson T Alfredsson L Kallberg H Calculating measures of biological interaction. Eur J Epidemiol 2005;20:575–9. 16119429 10.1007/s10654-005-7835-x

[30] Palmer TM Nordestgaard BG Benn M Association of plasma uric acid with ischaemic heart disease and blood pressure: mendelian randomisation analysis of two large cohorts. BMJ 2013;347:f4262. 23869090 10.1136/bmj.f4262 PMC3715134

[31] Du T Yuan G Zhang M Clinical usefulness of lipid ratios, visceral adiposity indicators, and the triglycerides and glucose index as risk markers of insulin resistance. Cardiovasc Diabetol 2014;13:146. 25326814 10.1186/s12933-014-0146-3 PMC4209231

[32] Fedchuk L Nascimbeni F Pais R Performance and limitations of steatosis biomarkers in patients with nonalcoholic fatty liver disease. Aliment Pharmacol Ther 2014;40:1209–22. 25267215 10.1111/apt.12963

[33] Petta S Di Marco V Di Stefano R TyG index, HOMA score and viral load in patients with chronic hepatitis C due to genotype 1. J Viral Hepat 2011;18:e372–80. 21692950 10.1111/j.1365-2893.2011.01439.x

[34] Matsuura F Yamashita S Nakamura T Effect of visceral fat accumulation on uric acid metabolism in male obese subjects: visceral fat obesity is linked more closely to overproduction of uric acid than subcutaneous fat obesity. Metabolism 1998;47:929–33. 9711987 10.1016/s0026-0495(98)90346-8

[35] Bagnati M Perugini C Cau C When and why a water-soluble antioxidant becomes pro-oxidant during copper-induced low-density lipoprotein oxidation: a study using uric acid. Biochem J 1999;340 (pt 1):143–52. 10229669 PMC1220232

[36] Sautin YY Nakagawa T Zharikov S Adverse effects of the classic antioxidant uric acid in adipocytes: NADPH oxidase-mediated oxidative/nitrosative stress. Am J Physiol Cell Physiol 2007;293:C584–96. 17428837 10.1152/ajpcell.00600.2006

[37] Ouyang X Cirillo P Sautin Y Fructose consumption as a risk factor for non-alcoholic fatty liver disease. J Hepatol 2008;48:993–9. 18395287 10.1016/j.jhep.2008.02.011 PMC2423467

[38] Lin SD Tsai DH Hsu SR Association between serum uric acid level and components of the metabolic syndrome. J Chin Med Assoc 2006;69:512–6. 17116612 10.1016/S1726-4901(09)70320-X

[39] Lu W Song K Wang Y Relationship between serum uric acid and metabolic syndrome: an analysis by structural equation modeling. J Clin Lipidol 2012;6:159–67. 22385549 10.1016/j.jacl.2011.11.006

[40] Babio N Martinez-Gonzalez MA Estruch R Associations between serum uric acid concentrations and metabolic syndrome and its components in the PREDIMED study. Nutr Metab Cardiovasc Dis 2015;25:173–80. 25511785 10.1016/j.numecd.2014.10.006

[41] Nakagawa T Hu H Zharikov S A causal role for uric acid in fructose-induced metabolic syndrome. Am J Physiol Renal Physiol 2006;290:F625–31. 16234313 10.1152/ajprenal.00140.2005

[42] Wortmann RL Gout and hyperuricemia. Curr Opin Rheumatol 2002;14:281–6. 11981327 10.1097/00002281-200205000-00015

