# Combined effect of obesity and uric acid on nonalcoholic fatty liver disease and hypertriglyceridemia: Erratum

**DOI:** 10.1097/MD.0000000000007231

**Published:** 2017-06-08

In the article, “Combined effect of obesity and uric acid on nonalcoholic fatty liver disease and hypertriglyceridemia”,^[1]^ which appeared in Volume 96, Issue 12 of *Medicine*, the funding information should appear as “This work was supported by grants from the National Natural Science Foundation of China (81570740)” rather than “no funding.”
